# Localized Tracheobronchial Transthyretin Amyloidosis Diagnosed After Hemoptysis: A Case Report

**DOI:** 10.1002/rcr2.70781

**Published:** 2026-10-06

**Authors:** Koichi Inoue, Yasumasa Tanaka, Masahiro Yanagi, Haruhiko Michimata, Yuta Nagahisa, Shun Kondoh

**Affiliations:** ^1^ Department of Respiratory Medicine Steel Memorial Muroran Hospital Muroran Japan

**Keywords:** ATTR amyloidosis, bronchoscopy, hemoptysis, localized amyloidosis, tracheobronchial amyloidosis

## Abstract

Pulmonary involvement in transthyretin amyloidosis (ATTR) is rare, and tracheobronchial involvement is particularly uncommon. We report a case of wild‐type ATTR amyloidosis presenting with hemoptysis in a 66‐year‐old woman with dyspnea. Chest computed tomography revealed bronchial wall thickening and infiltrative opacities. Bronchoscopy showed irregular mucosal lesions extending from the trachea to both bronchi. Histopathological examination of bronchial biopsy specimens showed Congo red‐positive deposits, and immunohistochemistry confirmed transthyretin, establishing the diagnosis of ATTR amyloidosis. Systemic evaluation and genetic testing excluded systemic and hereditary disease, leading to a diagnosis of localized wild‐type ATTR. Bronchial aspirate cultures yielded *Aspergillus fumigatus* and *Mycobacterium mucogenicum* , and antifungal therapy was administered for a suspected concomitant airway infection. This rare presentation of tracheobronchial‐localized ATTR amyloidosis underscores the importance of considering amyloidosis in the differential diagnosis of hemoptysis and the diagnostic role of bronchoscopy.

## Graphical abstract

Pulmonary involvement in transthyretin amyloidosis (ATTR) is rare, and tracheobronchial involvement is particularly uncommon. We report localized wild‐type ATTR presenting with hemoptysis and diagnosed by bronchoscopic biopsy showing Congo red‐positive transthyretin deposition.

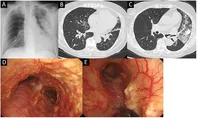

## 1 Introduction

Amyloidosis is caused by deposits of insoluble abnormal fibrillar proteins in organs, resulting in dysfunction. It is broadly classified into systemic and localized forms, the latter including disease confined to the lungs and airways. Pulmonary involvement is well recognized in immunoglobulin light‐chain amyloidosis [1, 2]; however, transthyretin (TTR)‐related amyloidosis (ATTR) rarely affects the lungs, and its clinical features remain poorly characterized. We report a case of wild‐type ATTR amyloidosis localized to the tracheobronchial tree and identified after hemoptysis.

## 2 Case Report

A 66‐year‐old woman presented to her primary physician with about 1 cup of hemoptysis and dyspnea. She was followed for hypertension and dyslipidemia and had prior left breast cancer surgery. There was no family history of amyloidosis. A nodular opacity in the left lower lung field had been noted about 10 years earlier and monitored over time. Because of decreased oxygen saturation and a new infiltrative opacity in the left mid‐to‐lower lung field on chest radiography (Figure 1A), she was referred emergently to our hospital.

**FIGURE 1:**
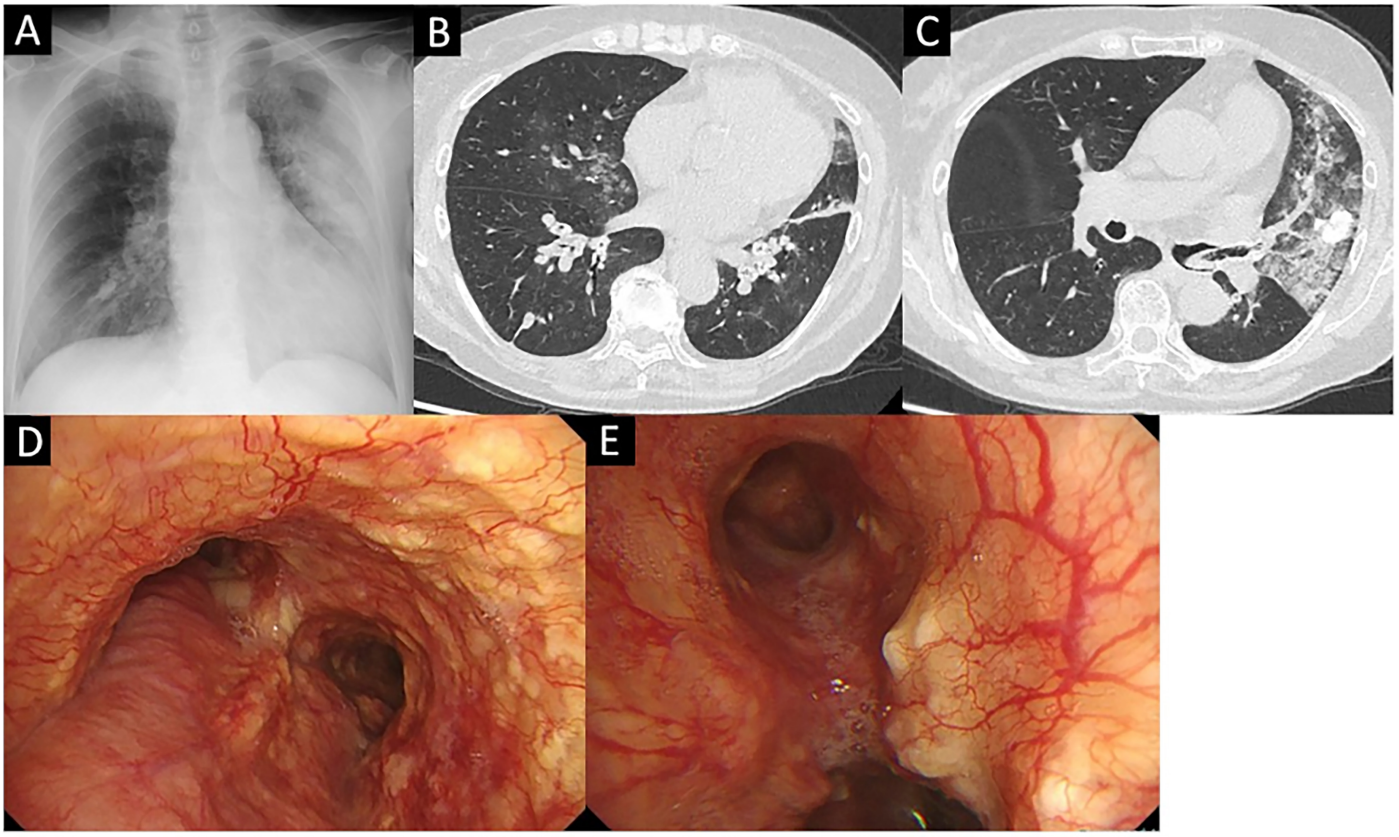
Chest radiography showed an infiltrative opacity predominantly in the left mid‐lung field, with the previously noted nodular opacity (A). Chest computed tomography showed bilateral bronchial wall thickening (B), retained material in the left main bronchus suggestive of blood‐tinged sputum, and infiltrative opacities with a calcified nodule in the left upper lobe (C). Bronchoscopy revealed irregular, bumpy mucosal lesions with a whitish‐to‐erythematous appearance (carina, D; bifurcation between the left upper and lower lobe bronchi, E).

Chest computed tomography (CT) showed bilateral bronchial wall thickening (Figure 1B), intraluminal material in the left main bronchus suggestive of blood clots, infiltrative opacities predominantly in the left upper lobe, and a calcified nodule in the left upper lobe (Figure 1C). White blood cell count and C‐reactive protein were not elevated. Biochemical and coagulation parameters were within normal limits. The interferon‐γ release assay for tuberculosis was negative, as were tumour markers.

After initial stabilization with hemostatic agents, bronchoscopy was performed. Irregular whitish‐to‐erythematous mucosal lesions were observed from the trachea to both bronchi (Figure 1D,E). The peripheral bronchi showed diffuse wall thickening with luminal narrowing. A biopsy was obtained from the right upper lobe bronchial orifice, and a bronchial aspirate was also collected. Histopathological examination of the bronchial biopsy specimen revealed amorphous, pale eosinophilic material in the subepithelial region (Figure 2A) and around vascular walls. Congo red staining was positive (Figure 2B). Potassium permanganate treatment was negative, and immunohistochemistry was positive for TTR (Figure 2C), confirming ATTR amyloidosis.

**FIGURE 2:**
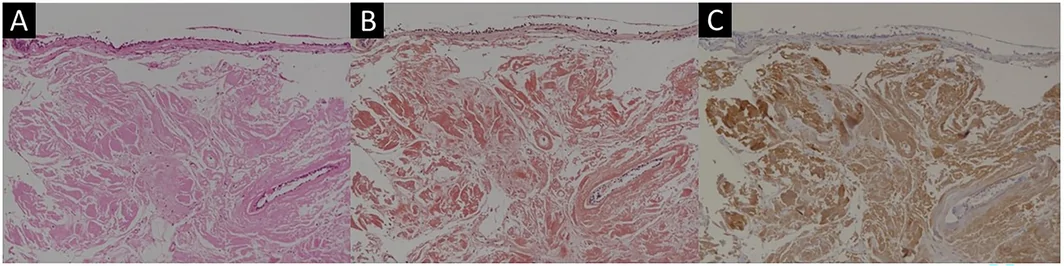
Histopathological findings of a transbronchial biopsy specimen. Histological examination confirmed ATTR amyloidosis (haematoxylin and eosin staining ×100, A; Congo red staining ×100, B; TTR immunostaining ×100, C).

Electrocardiography, echocardiography, and myocardial scintigraphy showed no findings suggestive of cardiac amyloidosis. There were no symptoms of sensory, motor, or autonomic nervous system dysfunction, and orthopaedic evaluation showed no evidence of carpal tunnel syndrome or spinal stenosis. Ophthalmologic examination also showed no apparent abnormalities. To exclude multiple myeloma and other plasma cell disorders, the hematologic evaluation included assessment of serum and urine monoclonal proteins and serum free light chains, as well as a bone marrow biopsy; no abnormalities were identified. Furthermore, no amyloid deposition was observed in gastrointestinal biopsy specimens obtained by endoscopy. Collectively, these findings showed no evidence of systemic amyloid involvement outside the lungs and bronchi. Peripheral blood sample analysis identified no pathogenic variants in the *TTR* gene. These findings established a diagnosis of wild‐type ATTR amyloidosis localized to the tracheobronchial tree.

Cultures of expectorated sputum and biopsy tissue were negative for bacteria, fungi, and acid‐fast bacilli, whereas bronchial aspirate cultures yielded *Aspergillus fumigatus* and *Mycobacterium mucogenicum* . Because amyloid deposition and airway infection could have contributed to hemoptysis, oral itraconazole was administered for approximately 1 year, and the mycobacterial infection was managed conservatively. Although a follow‐up bronchoscopy was not performed because the patient did not consent, a CT scan performed 1 year later showed reduced endobronchial secretions and improved pulmonary opacities despite persistent bronchial wall thickening (Figure 3A,B). The patient has had no recurrent hemoptysis, progression of airway lesions, or development of systemic disease.

**FIGURE 3:**
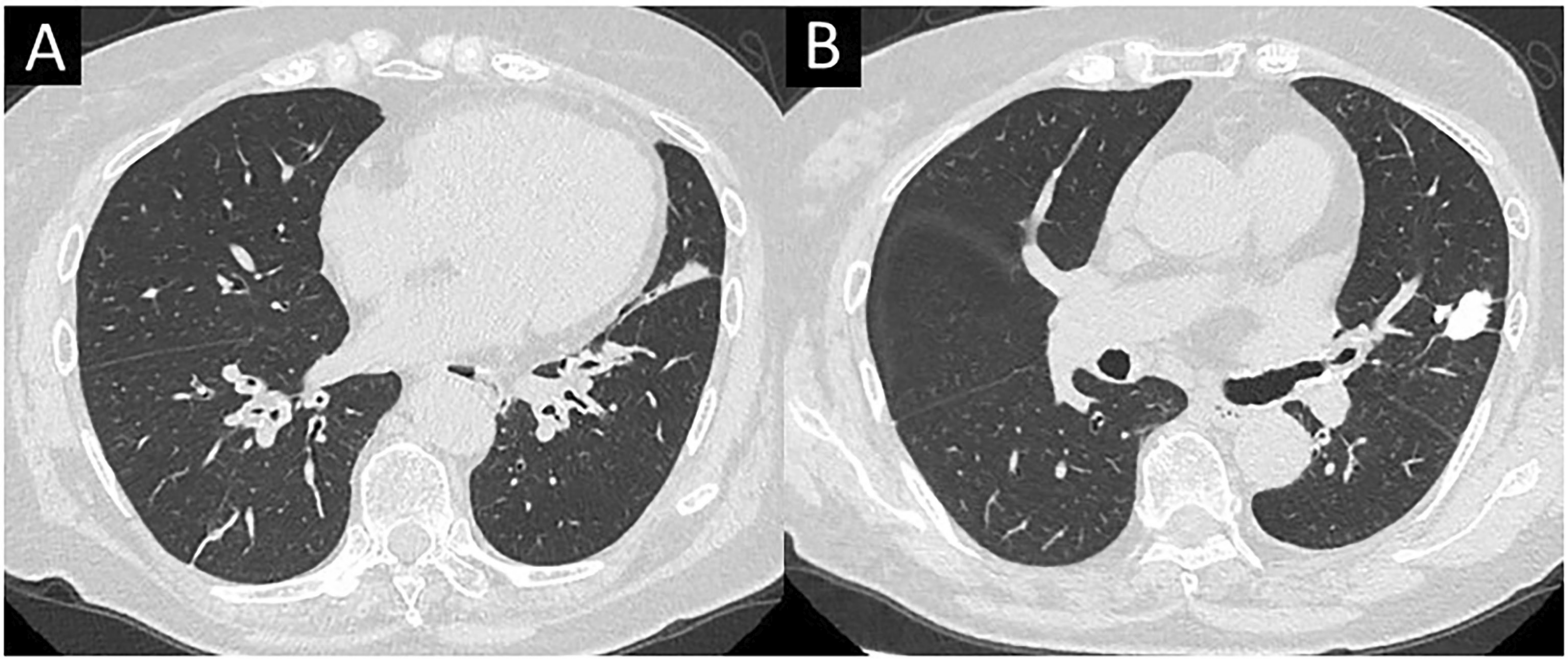
Chest computed tomography performed 1 year after the initial visit showed persistent bilateral bronchial wall thickening, with improvement in endobronchial secretions and pulmonary opacities (A, B).

## 3 Discussion

ATTR is broadly classified as hereditary ATTR, caused by pathogenic *TTR* gene mutations, or wild‐type ATTR, which is primarily age‐related. The heart, peripheral nervous system, and eyes are the most frequently affected organs. In contrast, pulmonary involvement is uncommon, and its pathophysiology remains unclear. Pulmonary amyloidosis is conventionally categorized into nodular, diffuse alveolar septal, and tracheobronchial forms [1]. In ATTR, the nodular pattern is reported most often, with radiologic findings such as calcified nodules, interlobular septal thickening, and cystic changes. However, tracheobronchial tree involvement has been described in only a few cases [2, 3]. In the present case, despite a calcified pulmonary nodule, imaging and bronchoscopic findings indicated predominant tracheobronchial involvement without evidence of systemic disease, supporting localized ATTR. This phenotype is exceptionally rare.

Dyspnea is the most common presenting symptom of pulmonary amyloidosis, followed by cough, weight loss, and chest pain; hemoptysis is relatively uncommon (approximately 7%) [2]. Tracheobronchial amyloidosis may cause secondary infections and bleeding through mucosal fragility and bronchial stenosis from submucosal amyloid deposition [4]. Although hemoptysis is uncommon, amyloidosis should be included in the differential diagnosis of hemoptysis. A prospective study of 606 patients with hemoptysis reported diagnostic yields of 77.3% with CT, 48.7% with bronchoscopy, and 83.9% with a combined approach [5]. These findings suggest that, in some cases, the cause of hemoptysis can be identified without bronchoscopy, such as when CT reveals cavitary or nodular opacities strongly suggestive of pulmonary tuberculosis and sputum testing is positive for *Mycobacterium tuberculosis* . However, when the cause of hemoptysis is not established by imaging or other noninvasive tests, or when airway lesions are suspected, bronchoscopy may provide additional diagnostic information. In the present case, a pathological diagnosis of ATTR amyloidosis was established by bronchoscopic biopsy. In addition, bronchial aspirate culture identified a concurrent airway infection that may have exacerbated hemoptysis associated with amyloid deposition. These findings informed the subsequent management strategy. Therefore, when the cause of hemoptysis remains unclear, bronchoscopy should be considered.

Although the patient has had no recurrent hemoptysis during follow‐up, patients with progressive airway stenosis or recurrent hemoptysis may require additional interventions. Disease‐modifying therapies for ATTR, including transthyretin stabilizers and gene‐silencing agents, are currently approved in Japan for cardiac and/or neurologic involvement; however, their efficacy in pulmonary disease remains unclear. For tracheobronchial amyloidosis, interventional approaches such as bronchoscopic debulking, laser therapy, and radiotherapy have been reported [1, 2, 4], but no standardized treatment has been established, and additional cases are needed.

In conclusion, we report a case of ATTR identified after hemoptysis onset. Although exceedingly rare, this case shows that tracheobronchial‐localized ATTR can occur. It also emphasizes the importance of including amyloidosis in the differential diagnosis of hemoptysis and highlights that bronchoscopy may provide diagnostic value, particularly when a definitive diagnosis cannot be established by imaging or other noninvasive examinations alone or when airway lesions are suspected.

## Author Contributions

**Koichi Inoue:** investigation, writing – original draft, conceptualization. **Yasumasa Tanaka:** writing – review and editing, supervision. **Masahiro Yanagi:** writing – review and editing, writing – original draft. **Haruhiko Michimata:** investigation. **Yuta Nagahisa:** conceptualization, investigation. **Shun Kondoh:** writing – review and editing, supervision.

## Funding

The authors have nothing to report.

## Consent

The authors declare that written informed consent was obtained for the publication of this manuscript and accompanying images using the consent form provided by the Journal.

## Conflicts of Interest

The authors declare no conflicts of interest.

## Data Availability

The data that support the findings of this study are available on request from the corresponding author. The data are not publicly available due to privacy or ethical restrictions.
